# Neurocognitive effects of repeated ketamine infusion treatments in patients with treatment resistant depression: a retrospective chart review

**DOI:** 10.1186/s12888-022-03789-3

**Published:** 2022-02-22

**Authors:** Danika Dai, Courtney Miller, Violeta Valdivia, Brian Boyle, Paula Bolton, Shuang Li, Steve Seiner, Robert Meisner

**Affiliations:** 1 Psychiatric Neurotherapeutics Program, McLean Hospital, 115 Mill St, Belmont, MA 02478 USA; 2 Department of Psychiatry, Harvard Medical School, Boston, MA USA

**Keywords:** Ketamine, Repeated intravenous infusions, Neurocognition, Treatment-resistant depression

## Abstract

**Background:**

Ketamine has emerged as a rapid-acting antidepressant in treatment-resistant depression (TRD) increasingly used in non-research, clinical settings. Few studies, however, have examined neurocognitive effects of repeated racemic ketamine infusion treatments in patients with TRD. In an effort to identify potential effects after serial infusions, we conducted a retrospective chart review to identify statistically significant changes in cognition in patient undergoing serial intravenous infusions; concomitantly, we examined baseline cognition as potential predictor of anti-depressant potential.

**Methods:**

Twenty-two patients with TRD were examined after they finished the induction phase of 8–10 repeated intravenous ketamine infusions and completed the assessments of their depressive symptoms (measured by the 16-item Quick Inventory of Depressive Symptomatology-Self Report Scale: QIDS-SR16) and cognitive function (measured by the Montreal Cognitive Assessment: MoCA) before the first and the last ketamine treatments.

**Results:**

Repeated ketamine infusions administered through an escalating dose protocol with 8–10 infusion sessions produced a 47.2% reduction response in depression; there was no evidence of impairment as reflected in MoCA testing. There was a moderate association between baseline cognition and antidepressant response with a Pearson correlation of 0.453.

**Conclusion:**

In this naturalistic sample of patients with TRD in our clinical service, repeated ketamine infusions significantly decreased depression symptoms without impairing cognitive performance. The baseline cognition may positively predict antidepressant responses of repeated ketamine treatment.

## Background

Major depressive disorder (MDD) is a common and disabling mental condition that affects up to one in five adults in the United States during their lifetime, and more than 300 million people worldwide at any given time, according to the World Health Organization [1, 2]. Furthermore, up to one-third of patients are considered to have treatment-resistant depression (TRD), defined as an insufficient response to two more adequate trials of antidepressant medications [3].

Ketamine, an N-methyl-D-aspartate receptor antagonist, was demonstrated to have a rapid antidepressant action in a randomized double-blind trial for the first time in 2000 [4]. Since then, emerging studies have indicated rapid and robust antidepressant effects of ketamine in adults with TRD [5–9]. A single subanesthetic (0.5 mg/kg) dose infusion of intravenous (IV) ketamine has rapid-acting and robust antidepressant effects in at least 50% of patients, however, the effects dissipate by day 10 to day 14 [10–14]. Patients not responsive to a single infusion of ketamine may improve with subsequent infusions, and improvement following a single infusion can be sustained by subsequent infusions [15–17]. As a sample, in one uncontrolled, open-label study [15], fourteen patients with TRD received IV 0.5 mg/kg-0.75 mg/kg ketamine at a frequency of two per week for entire six infusions. In a completer analysis, the response rate was only 7% after the first three infusions but 42% after all six infusions. The remission rate was 17% after six infusions. The findings of this study suggest that patients may require multiple ketamine sessions to respond.

Cognition plays a key role in recovery and functional outcomes in MDD [18]. Given known harmful effects of ketamine in cognition when used in cohorts struggling with ketamine use disorders, there has remained concern that serial administration of low dose ketamine could have the potential to negatively affect cognition. Human results of acute ketamine use on memory are mixed [19]. Results of studies on chronic ketamine use have suggested that individuals who had abused it for a long period of time could be more prone to experiencing serious neurocognitive impairment [20–22]. Furthermore, a single infusion of ketamine (0.4 or 0.8 mg/kg) induced dose-dependently impaired episodic and working memory and slowed semantic processing, recognition memory, and procedural learning, and infusion of analgesic doses (8–20 mg/h) in healthy volunteers was shown to produce significant deficits in cognition [23, 24]. On the other hand, Ning’s group noted no deterioration in cognitive function from six ketamine infusions at 0.5 mg/kg over 12 days [25, 26]. Instead, a single infusion of 0.5 mg/kg in TRD patients was seen to be slightly beneficial in attention and response control [27].

As concerns regarding ketamine and cognition have evolved, concomitant curiosity regarding the predictive value of baseline neurocognitive function has emerged [26, 28, 29]. Murrough et al. [28] have suggested individuals with TRD performing with lower neurocognitive function at baseline were more likely to obtain a positive antidepressant response from a single ketamine infusion. On the other hand, a study conducted by Bönke’s group indicated no significant correlation between baseline cognitive performance and a change in symptom severity, nor a correlation between a change in cognitive performance and antidepressant responses from six series ketamine infusion treatments [30].

In 2018, we began to treat patients with TRD by ketamine infusion in the clinical Ketamine Service at McLean Hospital. Over the years, we have refined a serial infusion ketamine protocol based on the evolving literature and our clinical experience. The protocol includes an induction phase and either a booster or maintenance phase. The induction phase consists of 8–10 treatments on a twice-weekly schedule with the IV ketamine dose initiated at 0.5 mg/kg over 40 min; thereafter, titration to response was permitted in a conservative fashion at the discretion of the ketamine team with the maximum dose up to 1.0 mg/kg.

In this real-world context, the investigation below seeks to 1) report the neurocognitive effects of repeated ketamine infusions in patients with TRD who have finished the induction phase through a retrospective chart review and 2) examine whether there is an association between cognition at baseline and antidepressant effects induced by repeated ketamine infusion.

## Methods

### Clinical procedure

DSM-5 Diagnostic Criteria were used to define patient with MDD. Ketamine treatment was offered to patients with severe and refractory MDD with at least two or more failures of anti-depressant treatment at adequate dosing, while patients with a history of psychosis, current substance use disorder, or uncontrolled medical illness, were not eligible for ketamine treatment. After psychiatric consultation and medical assessment, patients who were appropriate for ketamine treatment reviewed and signed a consent for ketamine treatment that emphasized that ketamine was not approved by the U.S. FDA for any psychiatric indication, and was provided off-label for depression, in addition to potential risks and benefits. At each visit, patients were evaluated and monitored by a staff psychiatrist, a nurse, and an anesthesiologist as needed.

Depression symptom severity was evaluated with the 16-item Quick Inventory of Depressive Symptomatology-Self Report scale (QIDS-SR16) that is scored on a scale of 0 to 27, with 0 representing a complete absence of depressive symptoms and 27 representing the most severe symptoms [31]. The QIDS-SR16 was administered before the first treatment and every subsequent visit and data were entered into Research Electronic Data Capture (REDCap) system.

Cognition was evaluated with the Montreal Cognitive Assessment (MoCA) [32]. The 30 item MoCA, a brief cognitive screening tool for cognitive impairment, is recognized as a sensitive measure of cognitive function that can capture declines in cognition over repeated administrations. The MoCA is scaled from 1 to 30, with higher MoCA scores indicating better cognitive function; a MoCA score < 26 indicates impaired cognitive function [32]. The MoCA includes six cognitive domains, including visuospatial abilities; language; combined attention, concentration, and working memory; executive function; short-term memory recall; and orientation to time and place, although subsequent consensus has emerged that the total score is most meaningful. To avoid learning effects, clinicians only administered MoCA test twice during the whole ketamine induction phase treatment: the first one was before the first ketamine treatment as a baseline and the second one was at the last treatment of induction phase which two tests usually were administrated 4–5 weeks apart. In addition, two different versions of MoCA tests were randomly utilized among three different versions (version 7.1, 7.2, 7.3) to further prohibit better performance due to learned effects. Studies had shown all three MoCA versions are largely equivalent and the test–retest reliabilities show that this score is suitable to monitor cognitive change over time [33]. MoCA scores were entered into REDcap system.

Eligible participants for ketamine infusions received an acute induction course consisting of 8–10 infusions twice weekly over 4–5 weeks. Most participants received the first dose of 0.5 mg/kg of ketamine with possible dose escalation up to 1.0 mg/kg contingent on patient tolerability to the index dose and patient’s response. In terms of dosing strategy, there is currently no established consistent and optimal dose of intravenous ketamine for TRD [34]. Nevertheless, small, randomized trials that compared different doses of ketamine suggest that generally, the preferred dose may be 0.5 mg/kg of body weight [35]. However, dose adjustments may be appropriate for specific patients [36]. A dose of up to 1 mg/kg may be suitable for patients not responsive to 0.5 mg/kg [15]. In terms of frequency, in most randomized trials, the drug was given only one time and the benefit appeared to diminish over the following week. We have been using a twice-weekly schedule, consistent with the finding from a study that indicated infused ketamine twice-weekly or thrice-weekly for up to six weeks led to an improvement of depression with the two dosing frequencies comparable [37]. All infusions were administered over a period of 40–45 min, and participants were monitored at the clinic for up to two hours following treatment.

During the treatments, other pharmacological and psychotherapeutic treatments were continued as part of the usual regimen. In the procedure, patients who experienced transient dissociative symptoms or anxiety during the infusions could receive either intravenous infusion or 1 mg of oral lorazepam to improve the tolerability of the infusion. Patients who experienced nausea could receive intravenous or oral ondansetron. Blood pressure, heart rates, oxygen saturation, and respiration rates were monitored at regular intervals (5 min, then 10 min, then each 15 min) during the infusion and for 30 min afterward, with contingency planning for additional monitoring as clinically appropriate. Patients with a clinically significant increase in blood pressure could receive intravenous or oral labetalol. Criteria for discharge readiness included a return to baseline mental status, absence of gait disturbance and nausea, and normal blood pressure. Any administration required the patient to be discharged to the care of an adult escort, and driving was not permitted in the evening post-administration until the following day.

### Statistical analyses

In the present report, patients with TRD who started ketamine treatment from July 2018 to May 2021 were included. This retrospective data analysis was approved by the Institutional Review Board (IRB) of Mass General Brigham. Patients were included in the final analyses if they completed the induction treatment phase (8–10 sessions) and had completed MoCA and QIDS-SR16 questionnaires measured before the first treatment and the last treatment. Data of MoCA and QIDS-SR16 were captured from REDcap system. Data of clinical and demographic characteristic were extracted from original clinical consultation notes and each assessment notes in EPIC, one of electronic medical record system.

A two-tailed paired t-test was used to compare changes in metric scores between baseline and post-treatment. A logistic regression analysis was used to determine whether initial cognition scores measured by MoCA predict antidepressant response measured by reduction of QIDS-SR16 between baseline and post-treatment from repeated ketamine intravenous infusions. Percentage of reduction of QIDS-SR16 was calculated using the following formula: {(baseline QIDS-SR16-post treatment QIDS-SR16)/post treatment QIDS-SR16}*100. All analyses were performed using IBM SPSS version 21 and a *p*-value of < 0.05 was considered statistically significant.

## Results

### Patient clinical and demographic characteristics

From July 2018 to May 2021, seventy patients finished the induction phase for 8–10 intravenous ketamine treatments. Among those patients, twenty-two patients completed their depressive symptoms measured by QIDS-SR16 and neurocognitive function measured by MoCA before the first and last treatment. Table 1, summarized below, presents the demographic characteristics of these patients with TRD.

**Table 1:** Clinical and demographic characteristics of these patients

Characteristics	Patients for ketamine infusion treatment (*n* = 22)
**Age in years (mean ± SD) **	37.86 ± 16.95
**BMI (mean ± SD)**	26.08 ± 6.12
**Gender: Female, n (%)**	14 (63.64)
**Marital status, n (%)**	
Never married/divorced	10 (45.45)
Married/partner	11 (50.00)
Unknown	1 (4.55)
**Race, n (%)**	
White	19 (86.36)
African American	0 (0)
Hispanic	0 (0)
Asian	2 (9.09)
Unknown	1 (4.55)
**Educations completed, n (%)**	
Grade 6–12 or graduated high school	6 (27.27)
Graduated 4-year college	10 (45.45)
Graduate/professional degree	6 (27.27)
Unknown	0 (0)
**Current employment status, n (%)**	
Full-time	8 (36.36)
Part-time	1 (4.55)
On leave	4 (18.18)
Retired	1 (4.55)
Unemployed	5 (22.73)
Student	3 (13.64)
**History of substance use disorder, n (%)**	
EtOH use	3 (13.64)
THC	7 (31.82)
Illicit substance use	3 (13.64)
**Current concomitant psychiatric disorder, n (%)**	
Anxiety	10 (45.45)
PTSD	5 (22.71)
OCD	2 (9.10)
ADHD	4 (18.18)
**Concomitant medications, n (%)**	
Antidepressant drugs	100
Mood stabilizer	8 (36.36)
Antipsychotics	10 (45.45)
Stimulants	6 (27.27)
Benzodiazepines	10 (45.45)
**History of suicidal attempts, n (%)**	6 (27.27)
**Treatments history of ECT, n (%)**	8 (36.36)
**Treatments history of TMS, n (%)**	7 (31.82)

The remaining sample of twenty-two participants was roughly 64% female and mean age was 37.86 years. The 86.36% and 2% of those participants were white and asian respectively while there were no African American or hispanic patients in this study. This may reflect racial disparities in mental health care due to socioeconomic inequities. All participants were diagnosed with TRD and most common psychiatric comorbidities in those participants were anxiety, followed by PTSD, ADHD, OCD. All participants were taking antidepressants in addition to receive ketamine infusion. 36% and 31% patients also had previous treatments of ECT and TMS respectively.

### The effect of repeated ketamine infusions on cognition measured by MoCA

All patients received the first dose of ketamine IV infusion at 0.5 mg/kg with possible dose escalation up to 1.0 mg/kg contingent on patient tolerability to the index dose and patient’s response. The average dose of ketamine at the last treatment for those twenty-two patients was 0.67 mg/kg with standard error at 0.03.

**Fig. 1:**
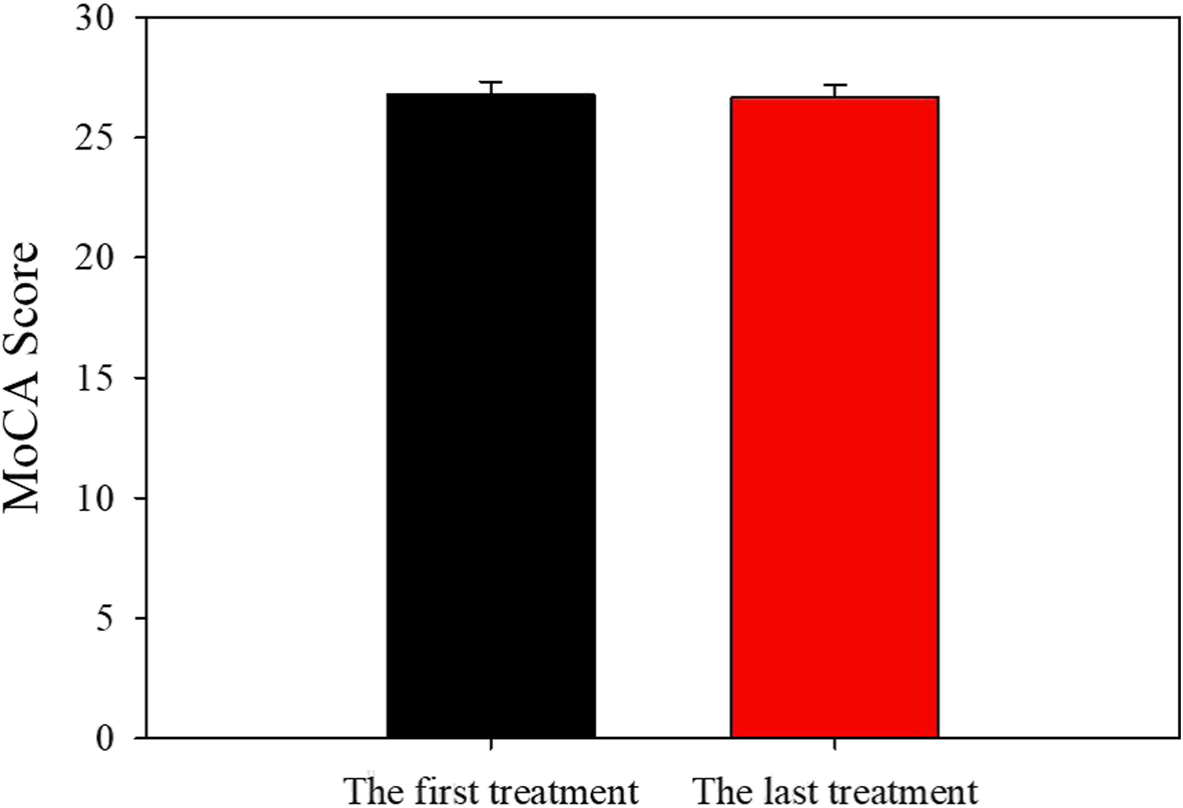
The effect of repeated ketamine infusions on cognition. The data are expressed as mean + S.E.M. There was no significance of difference between baseline cognition and cognition after repeated IV ketamine infusions: *P* > 0.05, (two-tailed paired t-test)

As shown in Fig. 1, the average MoCA score at baseline was 26.77 out of 30. The average MoCA score measured at the last treatment was 26.68 out of 30. There was no significant difference between baseline cognition and cognition after repeated IV ketamine infusions (*P* > 0.05).

### The effect of repeated ketamine infusions on depression measured by QIDS-SR16

**Fig. 2:**
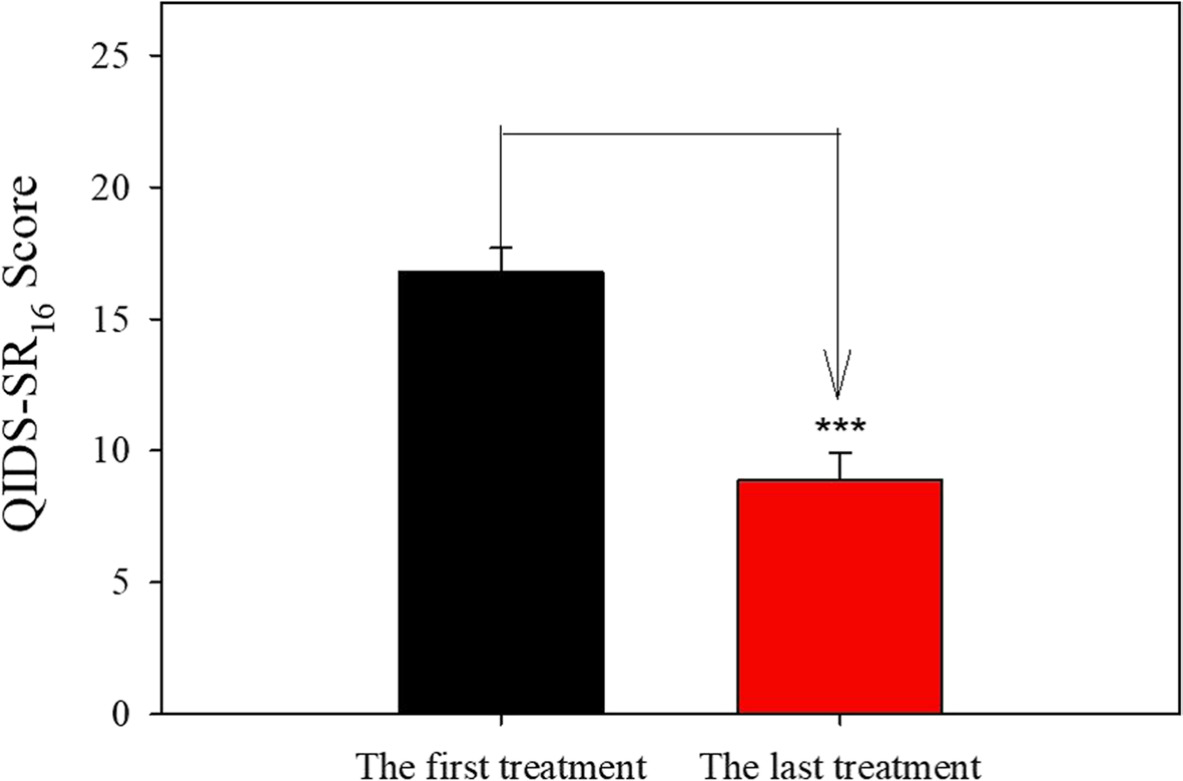
The effect of repeated ketamine infusions on depression. The data are expressed as mean + S.E.M. Significance of difference: ***P < 0.001, (two-tailed paired t-test)

As shown in Fig. 2, patients who have completed the induction phase after repeated ketamine infusions demonstrated significant improvement in depression measured by QIDS-SR16. Before the ketamine treatment, the average QIDS-SR16 was 16.77 which indicated severe depression. After repeated ketamine IV infusions, the average QIDS-SR16 was 8.86 which indicated mild depression. There was a significant reduction (47.17%) of QIDS-SR16 measured at the last treatment compared to the baseline QIDS-SR16 (two-tailed paired t-test *P* < 0.001). Among those 22 patients, QIDS-SR16 scores from six patients were in the range of 1–5 which indicated no depression.

### Baseline cognition measured by MoCA predicts ketamine’s antidepressant response measured by QIDS-SR16

A linear regression model was used to analyze the correlation between baseline cognition and antidepressant response after repeated ketamine infusions treatment. As shown in Table 2 A and B, the overall regression was statistically significant with R^2^ = 0.206, F (1, 20) = 5.176, *P* = 0.034 < 0.05. It was found that baseline MOCA significantly predicted the antidepressant responses of repeated ketamine infusions measured by the reduction QIDS-SR16 from baseline. This positive correlation effect was moderate with a Pearson correlation of 0.453.

**Table 2:** A and B Linear regression analysis of MoCA score at baseline and reduction of QIDS-SR16 from baseline

A: Model Summary						
Variables		Predictor: MoCA score				
		Pearson correlation			R Square	
Dependent variable: Reduction of QIDS-SR16 from baseline (%)		0.453*			0.206	
B: ANOVA						
	Sum of Squares		df	Mean Square		Sig *P* (2-tailed)
Regression	2523.8		1	2523.8		0.034* < 0.05
Residual	9752.1		20	487.6		
Total	12,275.9		21			

Significance of difference, *P < 0.05, (2-tailed)

## Discussion

We first examined the antidepressant effects of repeated 8–10 ketamine infusions at escalating dose protocol at our clinic. The results from this study suggest that patients with TRD who have completed the induction phase have demonstrated significant improvement in their depression, with a 47.2% reduction of their QIDS-SR16 score compared to their baseline. These results are consistent with other ketamine studies in similar clinical settings with non-randomized patients. Those studies indicate a response rate from 45–50% from either 6–9 infusions or 4-infusion protocol with 0.5 mg/kg of repeated intravenous ketamine treatments [8, 30]. In most randomized controlled trials, the response rate to intravenous ketamine ranged from 30 to 70% on day 1 [8, 38, 39].

Secondly, we examined the effects of repeated escalating doses of ketamine intravenous treatment on the cognitive function. To the best of our knowledge, this is one of few studies that have investigated the effects of repeated escalating doses of ketamine intravenous treatment on the cognitive function of patients with TRD and compared the relationship of baseline cognitive function with antidepressant response in the clinical practice setting.

The fear of cognitive impairment can be a major barrier to repeated ketamine treatment in clinical practice. Studies have indicated that a cumulative dose of ketamine leads to neurocognitive impairments, decreased hippocampal function, and BDNF, suggesting potential dose-, frequency- and duration-dependent effects on cognition with ketamine [21, 40, 41]. Higher doses, higher frequencies, or longer durations of ketamine may cause more serious neurocognitive problems. In our clinical practice, we have demonstrated that repeated ketamine intravenous treatment at escalating dose protocol with 8–10 infusion sessions did not impair cognitive performance. There was no significant change in MoCA scores between post-infusions and baseline. Compared to previous studies, which have indicated that a single ketamine infusion at 0.5 mg/kg improved specific cognition as measured by the go/no-go task and six ketamine infusions at 0.5 mg/kg improved verbal learning and speed of processing, we did not find that repeated ketamine treatment improved cognitive function as measured by MoCA. Possible explanations for this discrepancy could be due to a different dosing schedule and different cognitive tests [26, 27]. In our clinical procedure, the starting dose was 0.5 mg/kg of ketamine, with a possible dose escalation of up to 1.0 mg/kg contingent on patient tolerability to the index dose and patient’s response. In addition, we administered the MoCA test post 8–12 sessions, instead of a single session [27] or six sessions [26]. Finally, the MoCA test could be different compared to other cognitive tests in terms of its sensitivity.

Regarding the relationship between baseline cognitive function and antidepressant response from repeated intravenous ketamine infusion treatments, we found that the baseline cognition measured by MoCA positively correlates to antidepressant response measured by QIDS-SR16 reduction. A higher baseline MoCA score predicts a better antidepressant outcome. Although the study from Bönke’s group [30] indicated no significant correlation between baseline cognitive performance and a change in symptoms severity, our findings are consistent with the finding that individuals with better visual learning at baseline were likely to obtain antidepressant response over six ketamine infusions [26].

This study has several limitations. First, as this is a retrospective chart review, patients were followed naturalistically without randomization nor a well-controlled group. They could continue or change antidepressants and psychotherapy regimen according to recommendations from their treating psychiatrist. Potential confounding effects due to natural changes or other treatments, including changes in antidepressants and psychotherapy, cannot be ruled out. Second, in our clinical practice, there was no fixed dosing schedule. We chose the first dose of ketamine intravenous at 0.5 mg/kg, with a possible escalating dose of up to 1.0 mg/kg depending on response and tolerability. The sessions for the induction phase could range from 8–10, with the majority being 8 sessions. Third, the high drop-out rate observed in our sample may have been influenced by the cost of ketamine infusions, since this treatment is not currently covered by most insurance companies. Due to their unpredictable busy schedules, more than 50% of patients who had completed the induction phase have either refused to finish the post-treatment MoCA assessment or were not offered the assessment by staff members.

## Conclusion

In conclusion, in this naturalistic sample of patients with TRD, repeated ketamine intravenous infusions significantly improve depression, with 47.2% of reduction of their depression symptoms. Repeated ketamine intravenous treatment at escalating dose protocol with 8–10 infusion sessions did not impair cognitive performance. There was a moderate association between baseline cognition and antidepressant response from repeated intravenous ketamine treatments. A higher baseline MoCA score may predict a better antidepressant outcome.

## Data Availability

The data cannot be shared due to confidentiality issues. Shuang Li, (MD, PhD. at Psychiatric Neurotherapeutics Program, McLean Hospital, 115 Mill St, Belmont, MA, 02,478. Tel: 617.855.2364. Email address: Shuang.Li@MGH.HARVARD.EDU) should be contacted if someone wants to request the data from this study.

## Abbreviations

TRD: Treatment-resistant depression
MDD: Major depressive disorder
QIDS-SR16: 16-Item Quick Inventory of Depressive Symptomatology-Self Report Scale
MoCA: Montreal Cognitive Assessment
IV: Intravenous
